# Alginate-pectin microparticles loaded with nanoemulsions as nanocomposites for wound healing

**DOI:** 10.1007/s13346-022-01257-9

**Published:** 2022-12-13

**Authors:** Chiara Amante, Valentina Andretto, Annalisa Rosso, Geraldine Augusti, Stefania Marzocco, Giovanna Lollo, Pasquale Del Gaudio

**Affiliations:** 1 Department of Pharmacy, University of Salerno, Via Giovanni Paolo II, 132, 84084 Fisciano, SA Italy; 2 University of Lyon, Université Claude Bernard Lyon 1, CNRS, LAGEPP UMR 5007, 43 Bd 11 Novembre 1918, 69622 Villeurbanne, France

**Keywords:** Nanocomposite, Nanoemulsion, Alginate, Pectin, In situ gelling powder, Wound healing

## Abstract

This work combines natural polymers with nanoemulsions (NEs) to formulate nanocomposites as an innovative wound dressing. Spray-drying has been used to produce alginate-pectin in situ gelling powders as carriers for NEs loaded with curcumin (CCM), a model antimicrobial drug. The influence of NEs encapsulation in polymer-based microparticles was studied in terms of particle size distribution, morphology, and stability after spray-drying. NEs loading did not affect the size of microparticles which was around 3.5 µm, while the shape and surface morphology analyzed using scanning electron microscope (SEM) changed from irregular to spherical. Nanocomposites as dried powders were able to form a gel in less than 5 min when in contact with simulated wound fluid (SWF), while the value of moisture transmission of the in situ formed hydrogels allowed to promote good wound transpiration. Moreover, rheologic analyses showed that in situ formed gels loaded with NEs appeared more elastic than blank formulations. The in situ formed gel allowed the prolonged release of CCM-loaded NEs in the wound bed, reaching 100% in 24 h. Finally, powders cytocompatibility was confirmed by incubation with keratinocyte cells (HaCaT), proving that such nanocomposites can be considered a potential candidate for wound dressings.

**Graphical Abstract:**

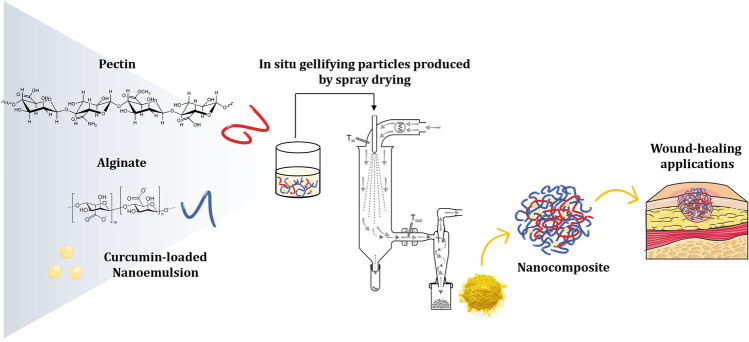

**Supplementary Information:**

The online version contains supplementary material available at 10.1007/s13346-022-01257-9.

## Introduction

The recent advancements in nanotechnology have provided suitable strategies to overcome most of the inconveniences associated with conventional dressings, such as bacterial infections, while offering cell-type specificity [1]. In particular, nanosystems have been combined with macro- and microstructures to develop nanocomposites aimed at increasing the stability of the encapsulated drug and promoting controlled release at the wound site [2–4]. The combination of different materials into a single formulation can enhance the specific properties of the single components leading to unique physicochemical and biological characteristics that the separate systems cannot achieve individually [5].

Different studies have reported the preparation of nanocomposites loaded with antibiotics (gentamicin and rifamycin), anti-inflammatory agents, or other biomolecules, to increase drug residence time in the wound sites, thus enhancing their activity [6, 7]. Also, composites containing gelatin, hyaluronic acid, and chondroitin sulfate associated with asiatic acid, ZnO, and CuO nanoparticles have been described as effective wound dressing scaffolds for second-degree burn wounds [8].

Along with nanocomposites, nanoemulsions (NEs) composed of an oily core stabilized by a surfactant shell [4, 9] have been described for wound healing applications [10]. NEs incorporating poorly water-soluble drugs in the oil droplet phase can be used to deliver lipophilic compounds to the superficial stratum corneum after topical application [11, 12]. However, since the viscosity of these systems is not high enough for a direct skin application, their encapsulation in a secondary structure such as polymeric scaffold is necessary [13]. Hydrogel-based nanocomposites are one of the most promising agents for wound healing thanks to their ability to favor skin adhesion maintaining a moist and cool environment [14].

Therefore, this work focused on the design of nanocomposites for direct wound application to deliver lipophilic drugs. To this aim alginate-pectin in situ gelling powders were embedded with curcumin-loaded NEs. In a previous work, we demonstrated that alginate with high mannuronic content and amidated pectin with a low degree of methylation were forming powders able to quickly gelify when in contact with wound fluids [15]. Sodium alginate with a high mannuronic content (over 65%) has been selected for its ability to form a soft and flexible hydrophilic gel through Ca/Na ionic exchange when in contact with the wound exudates and because it induces cytokine production by human monocytes, useful in the wound healing process [16]. To enhance the in situ gel-forming rate, low methoxyl grade pectin that gelifies ionotropically when in contact with bivalent cations present in the exudate has been associated with sodium alginate [17].

As a model drug for wound healing purposes, curcumin (CCM), 1,7-bis-(4-hydroxy-3-methoxyphenyl)-1,6-heptadiene-2,5-dione, has been selected as a model hydrophobic drug. CCM is a yellow phenolic pigment obtained from the rhizomes of turmeric (*Curcuma longa*), a tropical plant native to Southern Asia [18]. CCM, interfering with the inflammatory, proliferative, and remodeling phases and preventing oxidative damage, plays a great role in the treatment of wounds [19]. Moreover, recent studies showed that CCM possesses antibacterial activity against a broad range of microorganisms, including common bacteria that are detected in wounds: *Staphylococcus aureus*, *Escherichia coli*, and *Pseudomonas aeruginosa* [20]. Its bacteriostatic activity is linked to the interference with cellular processes by targeting DNA and proteins, cell wall and membrane damages, and inhibition of bacterial quorum sensing [21]. Here, nanocomposites were produced by mini spray-drying, and the influence of the addition of CCM-loaded NEs to the alginate-pectin powders was studied. Morphological analysis and particle size distribution, thermal behavior, fluid uptake ability, water vapor transmission rate, and viscoelastic properties of the powders were assessed. In vitro studies on the spontaneously immortalized human keratinocytes and cell line derived from adult skin (HaCat) have been performed to study the biocompatibility of selected powders.

## Materials and methods

### Materials

Medium-chain triglycerides, MCT (Miglyol^®^812), was purchased from Cremer Oleo GmbH & Co. KG (Hamburg, Germany). Polyoxyethylene (40) stearate (Myrj^®^52) and curcumin (CCM) were acquired from Sigma-Aldrich (St. Quentin-Fallavier, France). Oleoyl polyoxyl-6 glycerides (Labrafil^®^M1944CS) was supplied by Gattefossé (Saint-Priest, France). Sodium alginate Kelton LVCR from brown algae (1% viscosity 35 mPa s; mannuronic/guluronic ratio 70/30) was kindly donated by Dompè S.p.A (L'Aquila, Italy). Pectin Amid CF 025 D (amidated low methoxyl grade, degree of esterification 23–28%, degree of amidation 22–25%, molecular weight 120 kDa) was kindly offered by Herbstreith & Fox (Werder/Havel, Germany). Sodium chloride (NaCl) and phosphate buffered saline (PBS) tablets (pH 7.4) were acquired from VWR International (Fontenay-sous-Bois, France). Mycological peptone was purchased from Oxoid Ltd, Basingstoke, Hants, UK) and fetal bovine serum (FBS), qualified, heat inactivated from Gibco (Thermo Fischer Scientific, Brazil). Penicillin (10,000 U/mL) and 10 mg/mL streptomycin and Dulbecco’s Modified Eagle’s Medium (DMEM) were acquired from Euroclone (Euroclone, Milan, Italy). 3-(4,5-dimethylthiazol-2-yl)-2,5-diphenyltetrazolium (MTT) and DMSO were bought from Sigma (Sigma Aldrich, Milan, Italy). Dichloromethane, methanol, ethanol, acetonitrile (HPLC grade), formic acid, and sodium taurocholate hydrate 96% were purchased from Thermo Fisher Scientific (Illkirch, France). Milli-Q^®^ water was obtained using a Milli-Q^®^ Academic System from Merck-Millipore (Saint-Quentin-en-Yvelines, France).

### Preparation of curcumin-loaded nanoemulsions

Nanoemulsions (NEs) were prepared by emulsion phase inversion technique coupled with high stirring energy input as previously described [4]. Briefly, NEs composed of an oil core of medium chain triglycerides (MCT) were stabilized by a shell of surfactants, made of a mixture of polyoxyethylene (40) stearate (Myrj^®^52) and oleoyl polyoxyl-6 glycerides (Labrafil^®^M1944CS), hydrophilic and hydrophobic surfactants, respectively. To prepare the oil phase, the MCT oil core and surfactants were homogenized under magnetic stirring (750 rpm) using a thermostated bath at 80 °C. Then, the aqueous phase (PBS 5 mM pH 7.4), heated up to 80 °C, was added into the organic melt phase. The process of high stirring was then performed in two cycles of 10 min using a rotor–stator disperser (T25 digital Ultra-Turrax^®^ equipped with an S25N10G shaft, IKA^®^-Werke GmbH & Co. KG, Staufen, Germany) rotating at 11,000 rpm at 80 °C. The resulting colloidal system was cooled to room temperature under magnetic stirring for 30 min. CCM was added to the oil phase during NEs preparation. Nanoemulsions were prepared with a lipid concentration in the final phase of 142.86 w/v with 1.33% (w/w) of curcumin.

All studies and measurements presented in this work were performed in triplicate.

### Preparation of alginate-pectin powders and nanocomposites powders

To produce alginate-pectin (AP-11) powders and nanocomposites made of alginate-pectin with CCM-loaded NEs (AP-11-NE), mini spray-dryer (Mini Spray Dryer Büchi B 290, Büchi, Rungis, France) was used. AP solution was prepared by dissolving both polymers in Milli-Q under vigorous stirring for 1 h (500 rpm). The total concentration of polymers was set at 1% (w/v), and an alginate-pectin mass ratio of 1:1 was used. For the preparation of nanocomposites, AP-11-NE0.1, and AP-11-NE0.2, two different concentrations of NEs were added, to obtain a final concentration of 0.1% and 0.2% w/w, to the AP solution under a slight stirring (250 rpm) for 15 min before the spray-drying process. All formulations were processed with optimized parameters: aspirator 100%, drying airflow 560–580 L/h, air pressure 6 atmospheres, feed rate 3 mL/min, 120 °C inlet temperature, 65–68 °C outlet temperature, and nozzle diameter 0.7 mm.

The spray-dried powders were recovered and kept in closed vials to avoid moisture absorption. The process yield was calculated as the ratio between the amount of powders obtained and the total amount of processed material.

All powders were produced in triplicate.

### Size distribution and surface potential of nanoemulsions and nanocomposites

The size distribution and surface potential of the NEs were studied using the Malvern Zetasizer^®^ Nano ZS instrument (Malvern Instruments S.A., Worcestershire, UK). Particle size and polydispersity index (PDI) were determined by dynamic light scattering (DLS) diluting all samples with Milli-Q water to ensure the correct calculation of size distribution. Analyses were carried out at 25 °C with an angle of detection of 173°. The ζ–potential was calculated from the mean electrophoretic mobility measured for samples diluted in NaCl 1 mM. Measurements were performed in triplicate. The stability of NEs was followed for 28 days upon storage at 4 °C, measuring, at regular time points, particle size, PDI, and ζ–potential.

### Morphological analysis

The morphology of the nanocomposites was analyzed through scanning electron microscopy (SEM) at the “Centre Technologique des Microstructures” (CTμ) facility of the University of Lyon. SEM images were obtained with a FEI Quanta 250 FEG microscope with 10-kV accelerating voltage. Before microscopy, the powders were deposited on a flat steel holder and coated under a vacuum by cathodic sputtering with copper (10-nm layer).

### Static light scattering

Particle size distribution and mean diameter of powders produced by spray-drying were evaluated by Static Light Scattering Coulter LS 13,320 (Beckman Coulter, Inc., Fullerton, CA, USA). About 6 mg of AP-11, AP-11-NE0.1, and AP-11-NE0.2 was diluted in 6 mL of dichloromethane (DCM) and sonicated three times for 10 min. After sonication, some drops of each formulation were placed in DCM under constant stirring, using the micro liquid module making the average of three measurements for the sample. Results, calculated by instrument software using the Fraunhofer model, were expressed as mean diameter, and to evaluate the width of particle distribution span, Eq. 1 was used:

1 $$Span\;value=\frac{d90-d10}{d50}$$

where *d10*, *d50*, and *d90* indicate the volume diameters at 10^th^, 50^th^, and 90^th^ percentiles, respectively.

### Fluid uptake ability

Powder fluid uptake studies were conducted to evaluate the behavior of the powders when in contact with simulated wound fluid (SWF) containing 50% of FBS and 50% diluent composed of 0.1% (w/v) peptone, a peptic digest of animal tissue, and 0.9% (w/v) sodium chloride [22]. For fluid uptake ability studies conducted on dry powders, a Franz-type diffusion cell was used in an open configuration, without the donor chamber, with a total volume of 5 mL and a permeation area of 0.6 cm^2^.

About 8 mg of dried powder weighed on a microbalance (MTS Mettler Toledo, USA) was spread over a previously weighed HVLP nitrocellulose membrane (0.45 µm, Merck Millipore, Darmstadt, Germany). The membrane was in contact with a Franz cell (Hanson Research, USA), filled with SWF thermostated at 37 °C under stirring (200 rpm). At scheduled time intervals, the membrane with the sample was weighted, to establish the amount of fluid absorbed by the dry powder, and the Franz cell was refilled to maintain constant the volume of fluid during the entire experiment. Fluid uptake was calculated as the ratio between the weight of the gel and the weight of the dried powder producing the gel, using the following equation [23]:

2 $$Fluid \ uptake \ \left({\%}\right)=\frac{\mathrm{Ww}}{\mathrm{Wd}}\times 100$$

where *Ww* is the weight of the wet formulation and *Wd* is the weight of the dry formulation. All analyses were performed at least in triplicate.

### Water evaporation from hydrogel

Water vapor transmission rate (WVTR) to determine the moisture permeability of the wound dressing was performed as described by ASTM standard (ASTM Standard, 2010). Briefly, a 25-mm hydrogel disk, formed from each powder accurately weight and normalized respect the amount of AP when in contact with SWF, was mounted on the top of a plastic tube containing 20 mL of distilled water.

Tetrafluoroethylene was then used to cover the edge of the hydrogel disks to avoid boundary loss [24]. This system was kept in an incubator at 37 ± 0.5 °C with a humidity of 32 ± 0.2%. At defined time points, weight loss was noted and plotted against time.

WVTR was calculated as the ratio between the slope of the plot and the area of the disk normalized respect the amount of AP when in contact with SWF, by the following formula:

3 $$WVTR=\frac{Slope}{\mathrm{A}}$$

where *A* is the area of the sample in m^2^.

Water evaporation rate from in situ formed hydrogel was obtained as loss of weight over time by using the same procedure described above. After regular intervals, the weight was noted. The weight remaining was calculated by the following equation:

4 $$Weight \ increase=\frac{Wt}{W0} \times 100$$

### Residual water content

Water content contained in the powders was determined by Thermo Gravimetric Analysis (TGA) on NETZSCH TG 209F1 (NETZSCH-Gerätebau, Germany) using NETZSCH Proteus 6.1 software to analyze the data. About 5 mg of the powder was placed in ceramic crucibles and heated from 20 °C to 1000 °C at a heating rate of 10 °C/min, under a nitrogen atmosphere with a nominal gas flow rate of 30 mL/min.

In addition, the water content of the particles was quantified by Karl-Fischer titration using an 889 KF Coulometer (Metrohm Ltd., Herisau, Switzerland) equipped with an oven (860 KF Thermoprep). A solution of HYDRANAL (HYDRANAL™, Coulomat AG) was used as a titrant. The airflow rate was set at 100 mL/min and the oven temperature at 120 °C. The extraction time was set at 500 s, and the drift time was 10 µg/min.

Each powder was tested in triplicate.

### Differential scanning calorimetry

Differential Scanning Calorimetry (DSC) Q200^®^ instrument by TA Instruments (New Castle, DE, USA) was used to determine the thermal characteristics of powders. About 5–6 mg of each sample was placed in an aluminum pan (40 µL) perforated. Samples were subjected to both heating and cooling cycles in a range from + 20 °C to + 180 °C and from + 180 °C to − 80 °C, respectively. All DSC analyses were performed setting the flow rate at 10 ℃/min and the nitrogen atmosphere at 50 mL/min.

### Rheological measurements

The rheological properties of in situ gelled powders were evaluated through a MCR 302 rheometer (Anton Paar, Les Ulis, France) fitted with a 25 mm plate-plate geometry (PP25 with a diameter of 24.985 mm). Each powder was treated with 1 mL of SWF to form a gel in around 2 min. The distance between the plates was set at 0.5 mm, and the system was heated at 37 °C. Amplitude sweep tests were performed setting strain amplitude in the range of 0.01–200% and angular frequency at *ω* = 10 rad/s.

### Curcumin-loaded nanoemulsions: drug loading and encapsulation efficiency

The amount of CCM loaded in the NEs was quantified by UHPLC (ultrahigh-performance liquid chromatography) equipped with a PDA detector using the method reported in the literature [25, 26]. Pure CCM for the calibration curve and NEs were dissolved in methanol/acetonitrile (50:50). AP-11-NE powders were solubilized in methanol/acetonitrile and maintained under a stirring for 2 h to allow the complete dissolution of CCM from formulations. All the samples were vortexed for 5 min and filtered using a nylon filter of 0.22 μm (Whatman GmbH, Dassel, Germany) before injection in the UHPLC system. The UHPLC apparatus consisted of UHPLC Waters Acquity Arc Quaternary Solvent Manager-R and Waters Acquity Arc Sample Manager FTN-R, equipped with Waters Acquity UHPLC 2998 PDA Detector. CCM was detected using an RPC18 column (Kinetex 5 μm C18 100 Å, 150 × 4.6 mm, Phenomenex, Torrance, CA, USA), set at 30 °C, using acetonitrile and deionized water 0.1% formic acid (50:50) as mobile phase at a flow rate of 1 mL/min. The injection volume was 10 μL, the detection wavelength was 423 nm, and the total run time was 7 min. The chromatogram of CCM exhibited a characteristic peak at 4.7 min. The UHPLC calibration curThe amount of CCM ve was linear (R2 = 0.999) in the concentration range of 0.04–40 μg/mL. The method was validated according to ICH Q2(R1) guidelines. Detection and quantification limits (LOD and LOQ) were 0.00120 μg/mL and 0.0425 μg/mL, respectively.

The loading of CCM in NEs was calculated as the ratio between the CCM detected and the total weight of NEs, while the loading of NEs in AP powders was calculated through the quantization of CCM as the ratio between the CCM detected and the total weight of the powder. Encapsulation efficiency (E.E.) was calculated as the ratio of CCM detected to the amount of CCM initially loaded in the NE. The analyses were performed in triplicate.

### Nanoemulsions in vitro release

#### Solubility of curcumin

A saturated solution of CCM was prepared in SWF under stirring (750 rpm) at room temperature and left overnight to reach equilibrium. Then, the sample was centrifuged two times at 1200 RCF for 10 min, and the supernatant was collected and filtrated with a 0.22-μm nylon syringe filter (Whatman GmbH, Dassel, Germany). 200 μL of supernatant were mixed with 600 μL of methanol/acetonitrile (50:50), and the sample was injected into the UHPLC system for CCM detection.

#### In vitro release studies of nanoemulsions from AP-11-NE nanocomposites

The in vitro release of NEs from AP-11-NE nanocomposites was evaluated in SWF by cumulative study, in non-sink conditions, analyzing the CCM-loaded NEs by UHPLC as previously described. 2.5 mL of SWF were added on top of the AP-11-NE0.1 and AP-11-NE0.2 placed in a glass vial of 3 mL. 60 mg of powders of each sample were used. Vials were kept under a slight stirring at 37 °C. At predetermined time points, 200 μL were removed and replaced with fresh fluid. Samples were dissolved in 600 μL of methanol/acetonitrile (50:50) vortexed for 5 min and centrifugated for 2 min at 1200 RCF to remove the polymers. In the supernatants, 10 µg/mL of CCM as an internal standard was added to allow correct detection of the drug. The analyses were performed in triplicate.

### In vitro test

#### Cell culture conditions

Immortal keratinocyte (HaCaT) cell line derived from normal adult human skin was purchased from CLS Cell Lines Service GmbH (Eppelheim, Germany, accession number 300493) [27]. The cells were cultured with Dulbecco’s Modified Eagle’s Medium (DMEM) containing 10% (v/v) fetal bovine serum (FBS) and 1% (v/v) penicillin–streptomycin, at 37 °C in a humidified atmosphere of 5% CO_2_/ 95% air.

#### MTT test

The effect of the powders on the vitality of HaCaT cells was evaluated using a colorimetric assay with 3-(4,5-dimethylthiazol-2-yl)-2,5-diphenyltetrazolium bromide (MTT), as previously reported [28]. Briefly, HaCaT cells (5 × 10^3^ cells/well) were plated on 96-well plates and allowed to adhere for 24 h at 37 °C. After that, the medium was substituted with either fresh medium alone or one containing serial dilutions of powders (25, 50, 100 μg/mL) and incubated for 24, 48, and 72 h. 6-Mercaptopurine was used as a positive control. Then, 25 µL of MTT (5 mg/mL) was added, and the cells were incubated for further 3 h. Cells were then lysed, and the dark blue crystals were solubilized with 100 µL of a solution containing 50% (v/v) *N*,*N*-dimethylformamide, 20% (w/v) SDS with an adjusted pH of 4.5. The absorbance of the resulting solution in each well was recorded at 570 nm using an automated microplate reader (Titertek Multiskan MCC/340-DASIT, Cornaredo, Milan, Italy). Each sample was measured at least in triplicate, in three different experiments. The antiproliferative activity was calculated as % viability: 100 – [(OD treated/OD control) × 100], where OD is the optical density.

### Statistical analysis

Data are reported as mean ± standard deviation (SD) of at least three independent experiments, each in triplicate. Analysis of variance and Bonferroniʼs test were used for data analysis to perform multiple comparisons, using GraphPad Prism 8 (GraphPad Software). A *P* value less than 0.05 was considered significant.

## Results

### Nanoemulsions formulation, physicochemical characterization, and stability

NEs loaded with CCM were prepared by emulsion phase inversion technique coupled with a high stirring energy process, as previously described [29]. CCM was chosen as a hydrophobic model drug for its ability to enhance the wound healing process [30, 31].

Preliminary analyses conducted on NEs aimed at maximizing drug loading while maintaining the nanometric size (around 100 nm) and stability led to the formulation of nanosystems with a lipid concentration in the final phase of 142.86 w/v loaded with 1.33% (w/w) of CCM. Due to the lipophilic character of CCM, the drug encapsulation efficiency was very high (96.46 ± 5.66%), and the drug loading was 1.29 ± 0.10%.

**Fig. 1:**
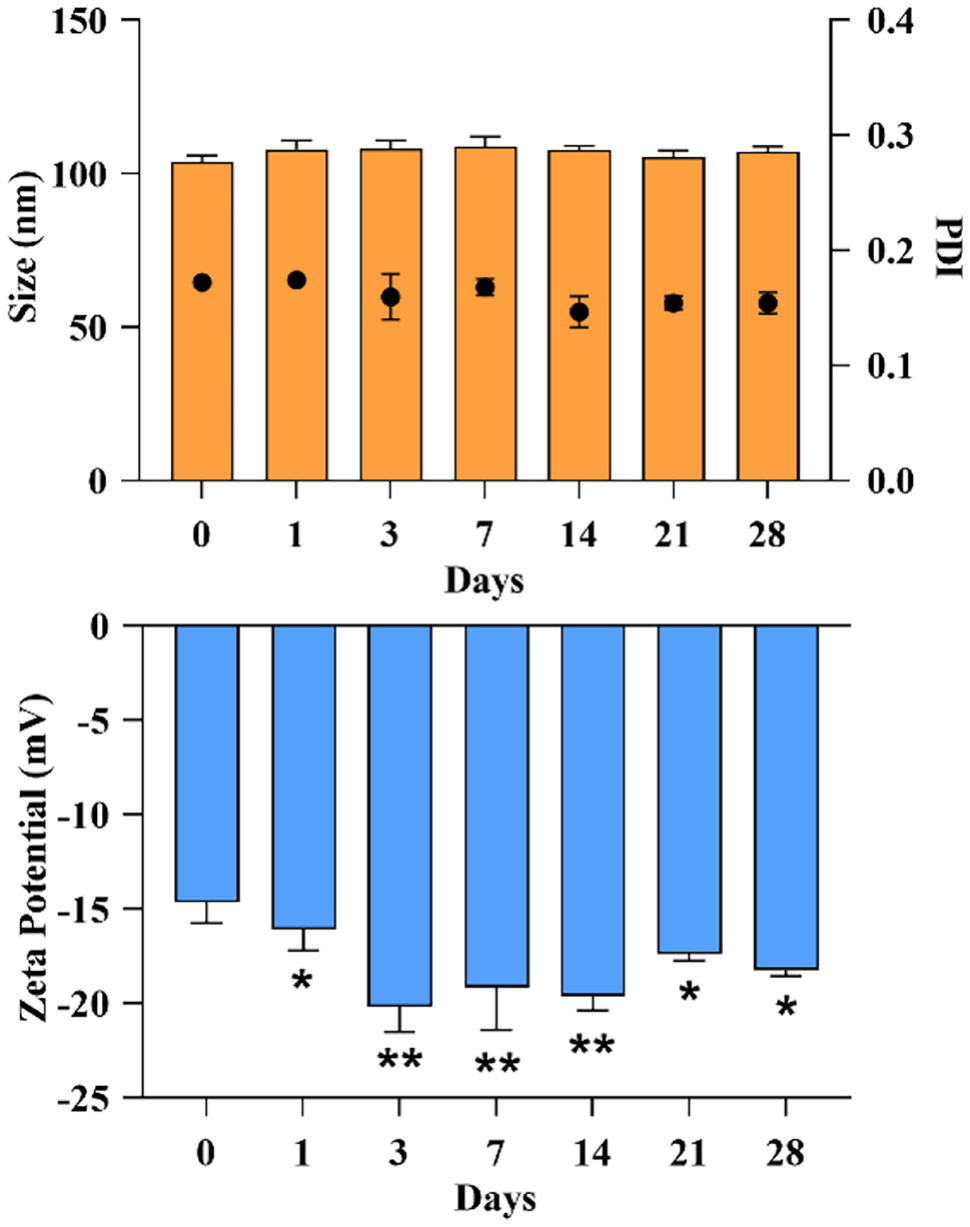
Stability study of NEs stored at 4 °C for 28 days. Data are shown as mean ± S.D., n = 3; ***, **, and * denote P < 0.001, P < 0.01, and P < 0.05, respectively, formulations vs control

The stability of NEs stored at 4 °C was followed over 28 days. Macroscopic aspects (presence of aggregates, cream formation, or color changes), physicochemical properties (particle size, polydispersity, and zeta potential), and drug leakage were evaluated. No sample degradation or changing of color was observed, and mean size, PDI, and surface potential remained stable during the studied period (Fig. 1). In addition, the amount of encapsulated CCM remained unchanged at the storage condition of 4 °C as confirmed by UHPLC analysis.

### Production and physicochemical characterization of nanocomposites

Once assessed the stability and encapsulation efficiency of CCM in NEs, an original nanocomposite composed of an alginate-pectin blend with NEs was produced using spray-drying. Based on previous work [15], alginate-pectin (AP) powder in a ratio 1:1 was chosen as a carrier (AP-11). While total polymers concentration was fixed at 1% (w/v), two different concentrations of NEs were added to obtain a final concentration in nanocomposites of 0.1% and 0.2% w/w. The main characteristics of nanocomposites were reported in Table 1. Both nanocomposites AP-11-NE0.1 and AP-11-NE0.2 were characterized by relatively high process yield without difference to microparticles AP-11. However, the increase of the NEs concentration determined a reduction of E.E., ranging from 57% for AP-11-NE0.1 to 50% for AP-11-NE0. 2.

**Table 1:** Main properties of alginate-pectin microparticles and nanocomposites

**Sample**	**Polymer concentration** **%** **(w/w)**	**NE** **%** **(w/w)**	**Process yield** **(%)**	**Drug content (%)** ± **SD**	**E.E.** **(%) **± **SD**
**AP-11**	1	-	67.0	-	-
**AP-11-NE0.1**	1	0.1	65.0	0.08 ± 0.00	57.60 ± 3.89
**AP-11-NE0.2**	1	0.2	65.1	0.14 ± 0.00	50.47 ± 0.59

The stability of the NEs after spray-drying was assessed by monitoring their physicochemical features after encapsulation in AP microparticles. After solubilization of microparticles in water, the complete dispersion led to the liberation of nanoencapsulated material with an increase in NEs diameter in NE0.1 and NE0.2 (viz., 0.1% and 0.2% nanoemulsions loaded in AP particles) from 103 to 205 nm and 238 nm, respectively. As shown in Table 2, PDI also showed an increase from 0.2 to 0.3 or to 0.4, respectively. Moreover, the ζ-potential of the NEs-loaded AP microparticles moved from a slightly negative value to a highly negative value (from − 15 to − 21 or − 29 mV). On the contrary, ζ-potential for AP-11-NE0.1 and AP-11-NE0.2 remains unchanged compared to AP blank particles. These results suggest a physical interaction between polymers and NEs.

**Table 2:** Physicochemical properties of NEs before and after encapsulation in AP particles, AP-11 microparticles, and moisture content of AP-11, AP-11-NE0.1, and AP-11-NE.0.2

	**Size (nm) ± SD**	**PDI**	**ζ–potential (mV) ± SD**	**H**_**2**_**o % (w/w) ± SD**
**NEs**	103.90 ± 2	0.2	-15 ± 1	-
NE0.1*	205.13 ± 2	0.3	-21 ± 2	-
NE0.2*	238.97 ± 5	0.4	-30 ± 2	-
**AP-11**	3517 ± 90	0.3	-52 ± 2	9.27 ± 0.04
**AP-11-NE0.1**	3338 ± 65	0.2	-51 ± 4	6.07 ± 0.05
**AP-11-NE0.2**	3325 ± 79	0.3	-50 ± 3	5.54 ± 0.09

*NE.01 and NE.02 represent the nanoemulsions released by a spray-dried particle, AP-11-NE0.1 and AP-11-NE0.2, respectively

The water content of the spray-dried powders (Table 2), evaluated by Karl Fisher titration, showed that the addition of NEs to AP-11 polymers led to a reduction in the amount of water entrapped during the spray-drying process, probably due to the added hydrophobic character.

**Fig. 2:**
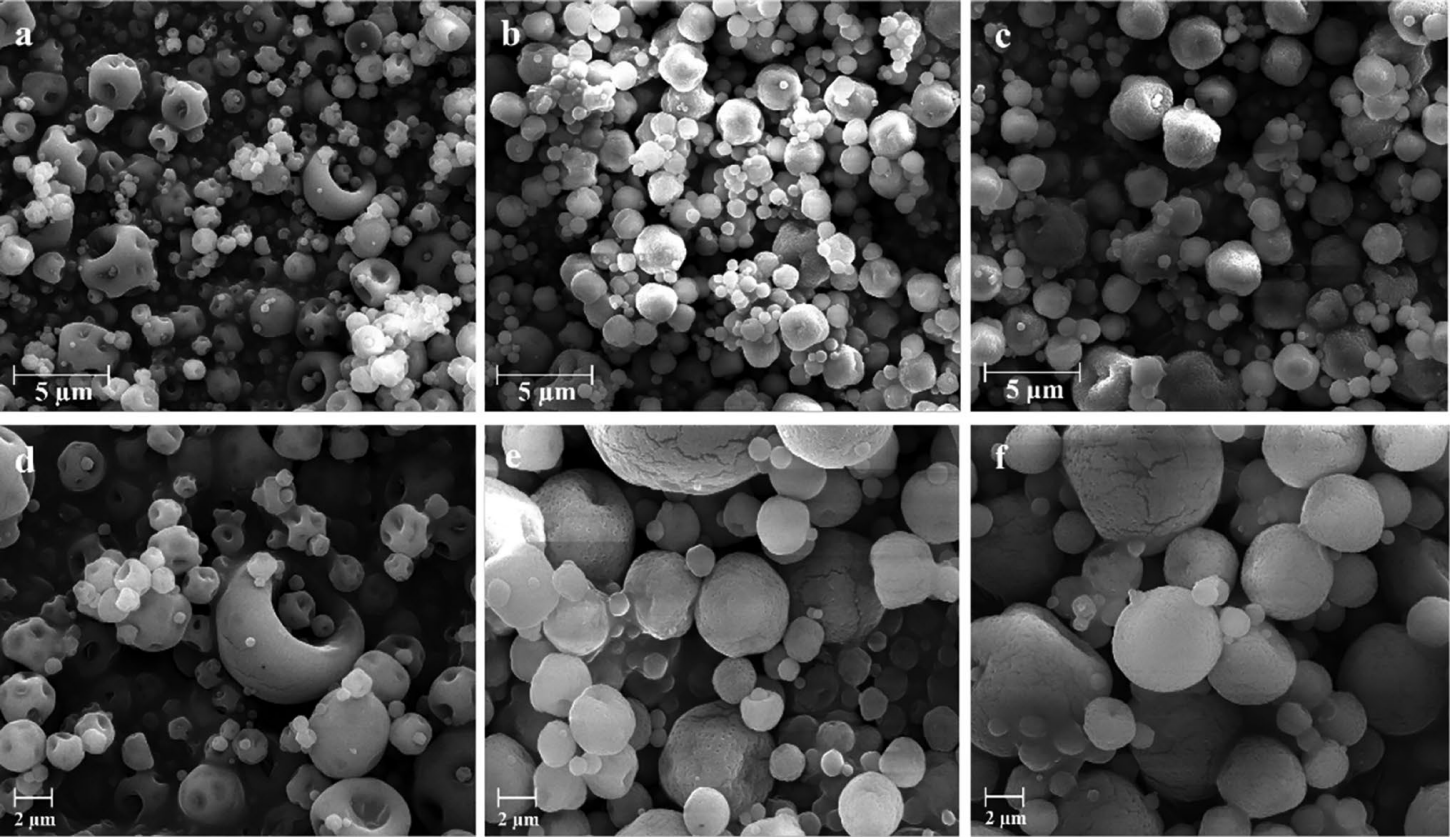
SEM microphotographs at two different magnifications of AP-11 (**a**, **d**), AP-11-NE0.1 (**b**, **e**), and AP-11-NE0.2 (**c**, **f**)

Morphology, as well as the dimensional distribution of microparticles, was affected by NEs content. AP-11 presented a morphology “deflated balloons” (Fig. 2a, d) which, in the nanocomposites, has been moved to spherical shapes with pores and small cracks due to the presence of NEs on the surface of the microparticles (Fig. 2b, c, e, f).

Moreover, dimensional distribution analyses performed by static laser scattering revealed that all the microparticles present a single peak particle size distribution with span values less than 2, with a mean diameter of about 3.5 μm, in accordance with SEM analyses.

**Fig. 3:**
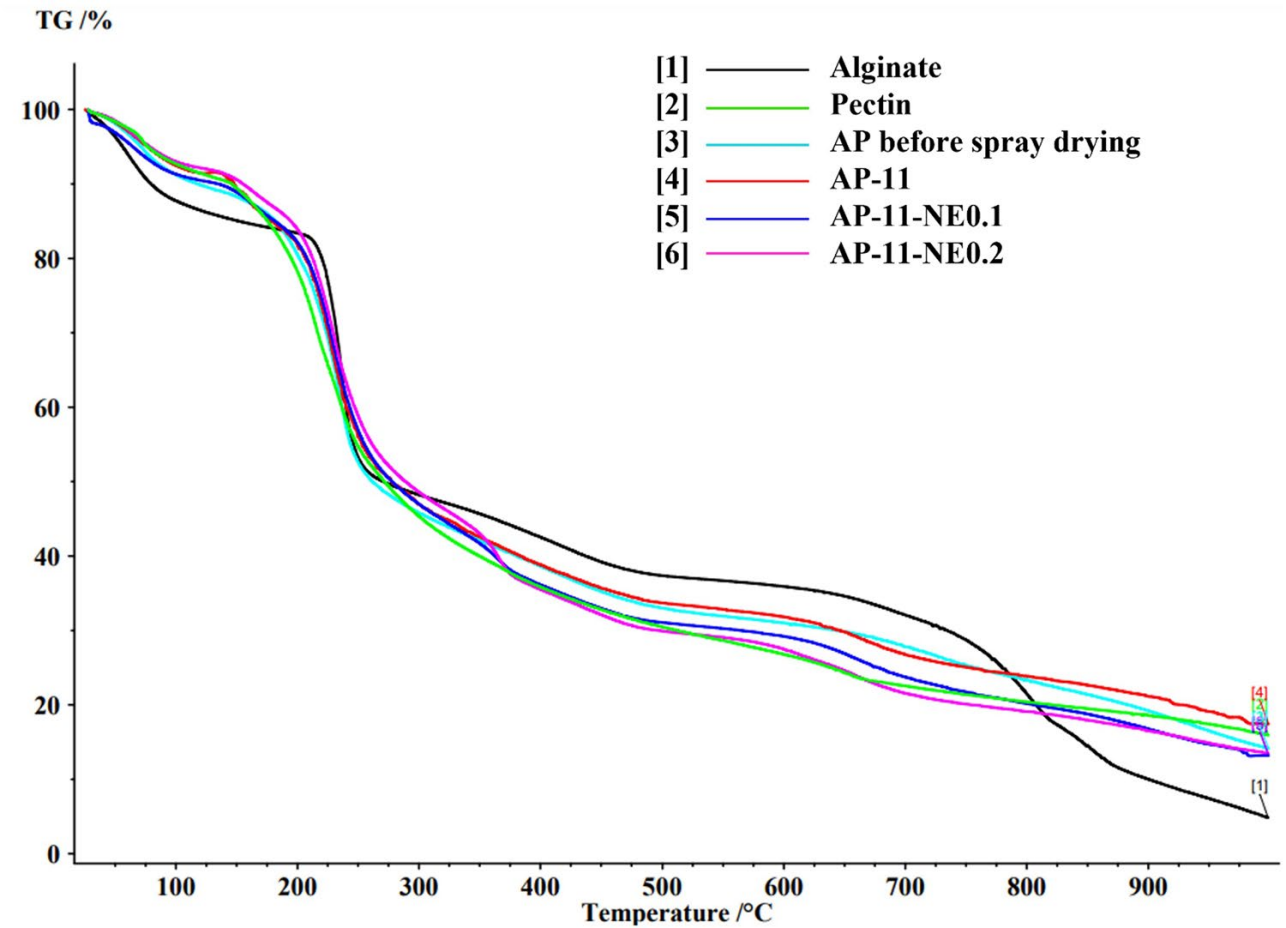
Thermogravimetric curves of alginate raw material (black), pectin raw material (green), AP before spray-drying (light blue) AP-11 (red), AP-11-NE0.1 (blue), and AP-11-NE0.2 (violet). (Color figure online)

The thermal properties of the materials were analyzed using both TGA and DSC. Figure 3 shows the TGA profiles of alginate and pectin polymers in comparison with those of the different microparticles. The degradation profile of alginate has shown a three-stage process: below 150 °C, the weight loss is associated with humidity release while at 237 °C and 750 °C, it is correlated to the rupture of chains, fragments, and monomers [32]. Differently, pectin presented two main weight loss steps. The degradation patterns of the other samples were very similar to alginate and pectin raw material with a weight loss below 150 °C due to the removal of moisture, followed by a sharp weight loss at 230 °C due to the degradation of the polymer backbone.

**Fig. 4:**
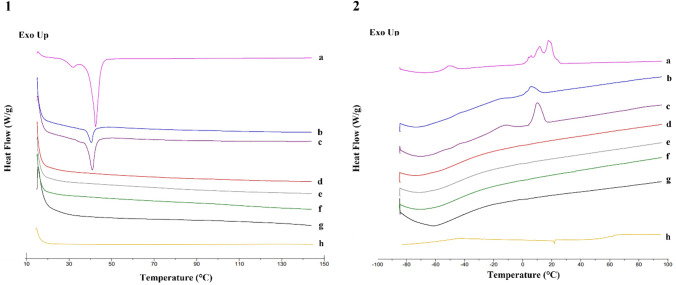
DSC thermograms of NEs (**a**), AP-11-NE0.1 (**b**), AP-11-NE0.2 (**c**), AP in combination before spray-drying (**d**), AP-11 after spray-drying (**e**), pectin raw material (**f**), alginate raw material (**g**), and curcumin raw material (**h**). Panel 1 represents the heating cycle from + 20 °C to 180 °C while panel 2 cooling performed from + 180 °C to − 80 °C both at 10 °C/min

The state of the shell (crystalline or amorphous) of the NEs loaded with curcumin and of the powders was investigated using DSC (Fig. 4). NEs (after water evaporation) showed a melting peak (Fig. 4-1a) at around 48 °C and a second peak at lower temperatures (below 40 °C) suggesting the presence of a second crystalline phase, ascribable to a polymorphic form of the stearic acid of polyoxyethylene (40) stearate resulting from its mixing with oleoyl polyoxyl-6 glycerides and MCT excipient [33]. In addition, a broad crystallization peak from 0 °C to 22 °C upon cooling and a second melting peak at − 50 °C (Fig. 4-2a) referred to as MCT polymorphism was present [33, 34]. AP-11-NE powders were analyzed to estimate the influence of the drying process on the structure of NEs loaded. After the water evaporation, the melting and crystallization peaks of polyoxyethylene (40) stearate were visible according to the amount of NEs loaded in the powders (Fig. 4b, c). On the contrary, alginate and pectin did not show any signal (Fig. 4d, e, f, g). Also, curcumin (Fig. 4h) did not exhibit any signal because its typical melting is visible at around 180 °C [35], outside the range of interest.

**Fig. 5:**
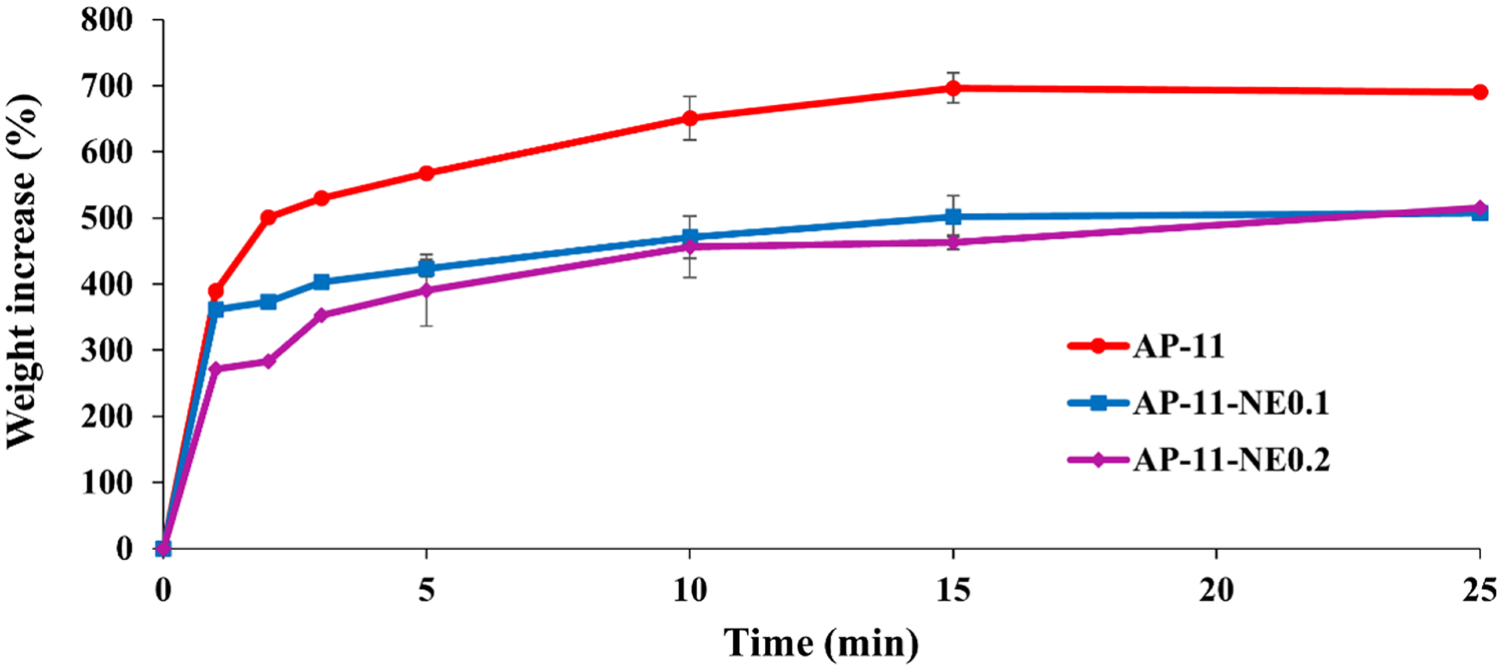
Simulated wound fluid uptake of AP-11, AP-11-NE0.1, and AP-11-NE0.2

To evaluate the influence of NEs on the powders ability to become a gel in situ, AP-11 and AP-11-NEs were placed in contact with simulated wound fluid (SWF), and, at scheduled time points, the increase in weight was calculated. Figure 5 shows that AP-11 became gel in less than 5 min increasing almost 7 times its weight after 15 min. As expected, a different trend was observed for AP-11-NEs. The swelling was very quick, but the water uptake was lower due to the presence of NEs which enhanced the hydrophobicity of the systems, as confirmed also by Karl Fisher’s analysis.

**Fig. 6:**
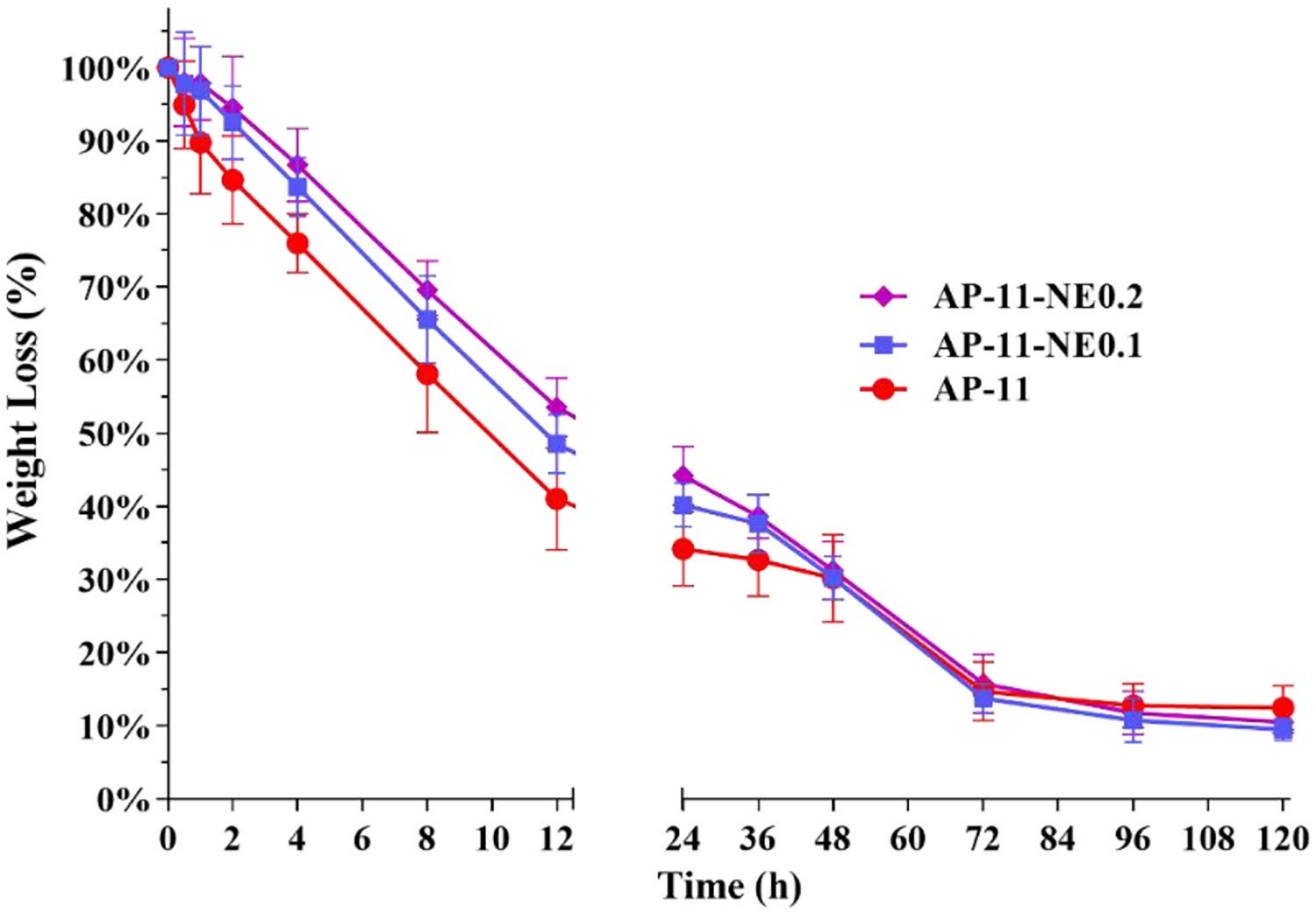
Weight loss from hydrogels made on alginate-pectin and alginate-pectin loaded with nanoemulsions, after 120 h

Water vapor transmission rate (WVRT) of the in situ formed hydrogels was measured to evaluate if the wound dressings are able to maintain proper moisture on the wound bed. All hydrogel formulations presented WVTR values between 85 and 89 g/m^2^h resulting then as an adequate barrier able to protect the wound while promoting good transpiration. The rate of water evaporation from hydrogels was measured to evaluate their ability to retain the water within the dressings over time even when exudate is not poured out from the wound anymore. Figure 6 shows the fluid loss evaluated as the weight decrease of the different formulations after the removal of SWF. It can be observed that the loss of water rapidly increased in the first 12 h, whereas after 72 h, there was almost no water loss from the hydrogels even though hydrogels were not completely dried yet. AP-11 showed that approximately 12% of water was retained, while AP-11-NE0.1 and AP-11-NE0.2 retained 10% and 11% water, respectively. It suggests that AP hydrogel, also when loaded with NEs, loses most of its water content in a short time, but can retain almost a proper amount of humidity even when exposed to air in dry conditions.

**Fig. 7:**
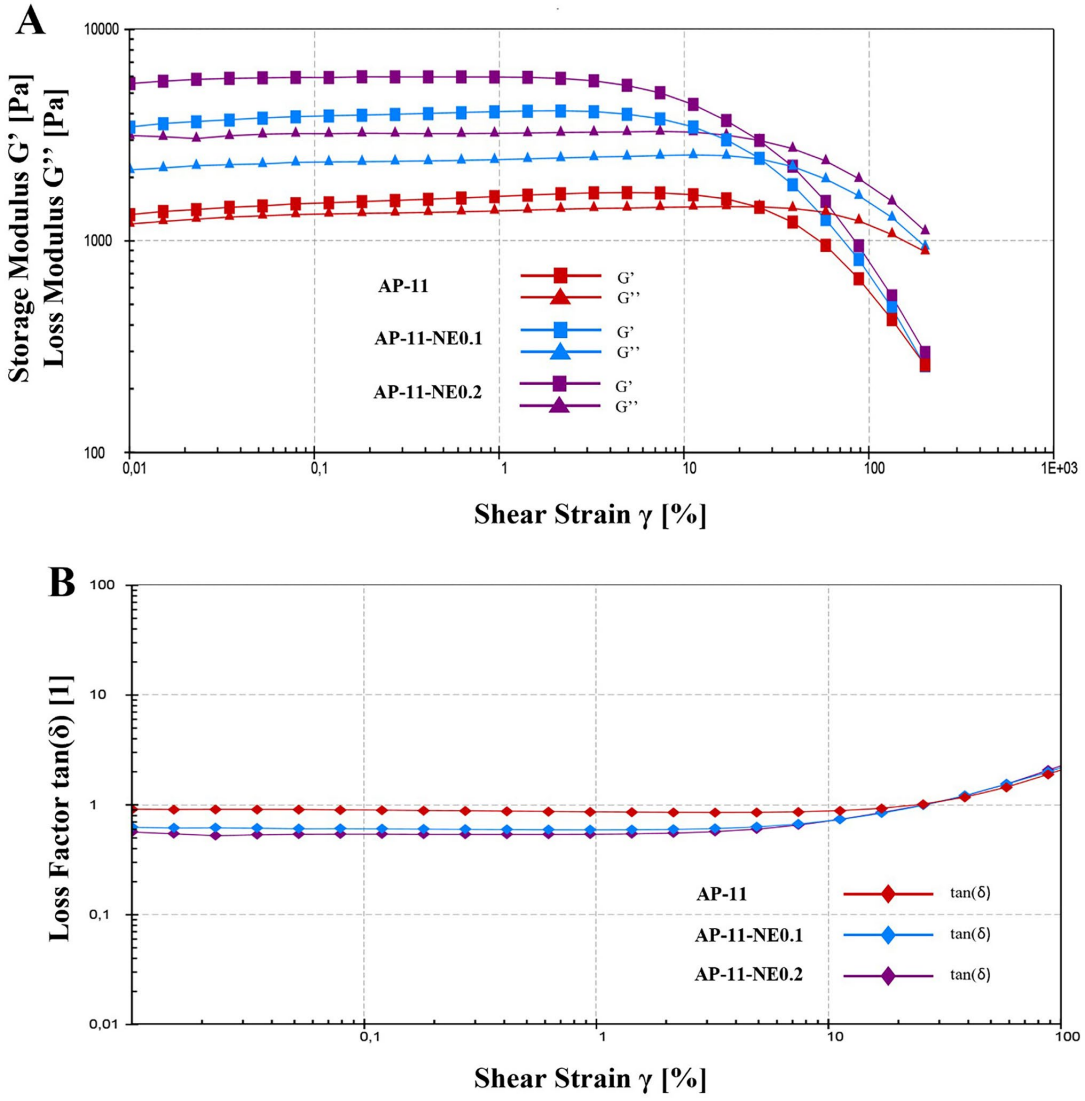
Rheological characterization of AP-11, AP-11-NE0.1, and AP-11-NE0.2. Storage G' and loss G" moduli evaluated via amplitude sweep test (Panel **A**) and strain dependent tangent of loss factor (Panel **B**)

The in situ gelled powders viscoelastic properties were assessed through rheological measurements performing an amplitude sweep test, which depicts the variation of dynamic storage modulus (G′) and loss modulus (G″) depending on the shear strain (γ). All formulations (Fig. 7) showed that G′ was much higher than G″. For the AP-11-NEs, an increase in the gap between the storage and loss modulus was reported, according to the NEs concentration, demonstrating the prevalent gel-like behavior. This pattern can be better explained through the loss factor that represents the ratio between viscous and elastic components (purely elastic, *δ* = 0; purely viscous, *δ* = 90) [36]. For the powder loaded with NEs, the loss factor decreased (Fig. 7 panel B) indicating an increase in structure formation and gelation.

### In vitro release study

**Fig. 8:**
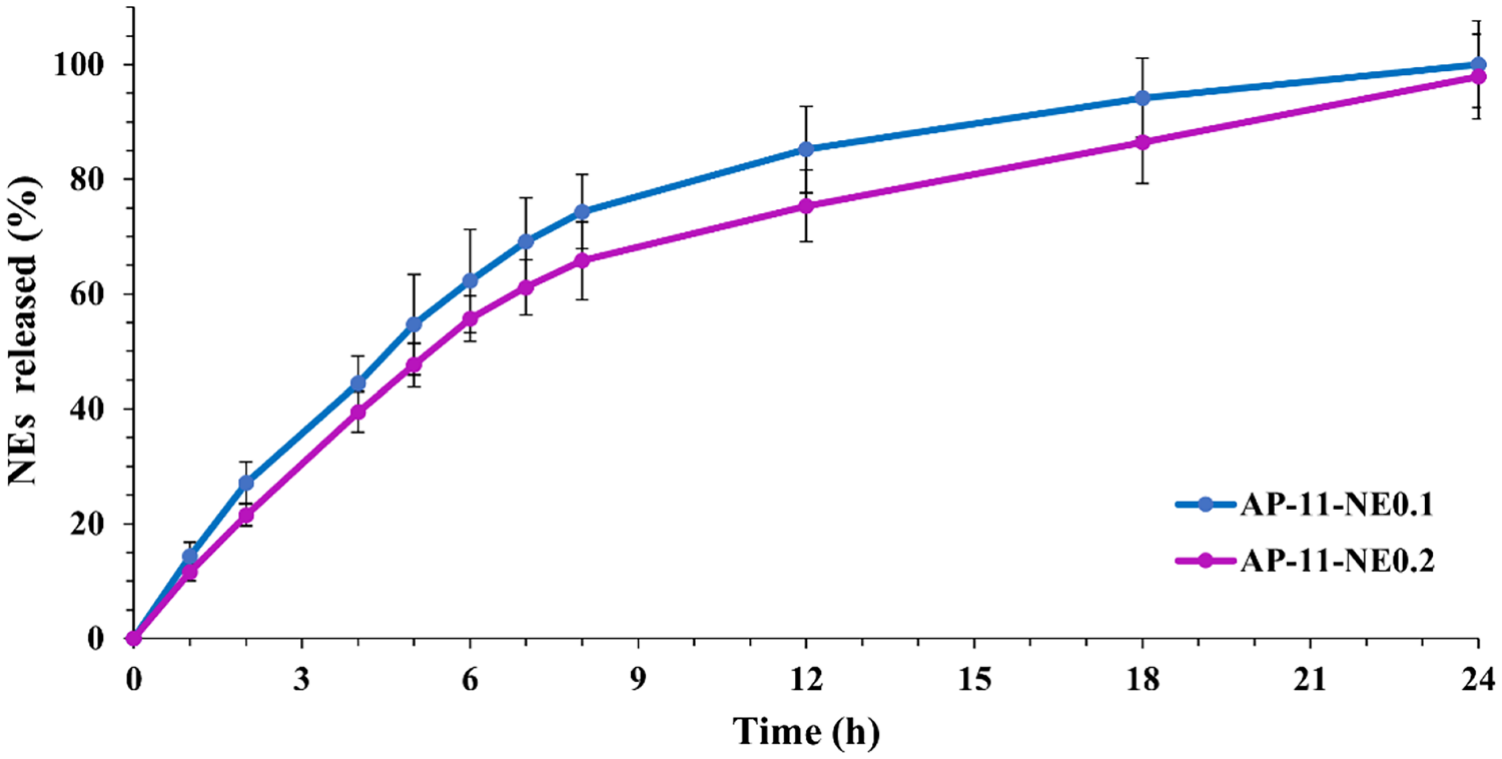
Nanoemulsions released from the nanocomposite AP-11-NE0.1 and AP-11-NE0.2 performed in simulated wound fluid

In vitro release studies of the NEs from nanocomposites were carried out in SWF. The study was carried out in non-sink conditions, and a cumulative release study was performed. All nanocomposites showed a similar profile. AP-11-NE0.1 released about 75% of the encapsulated NEs after 8 h, followed by a prolonged release up to 24 h reaching 100% of NEs release, whereas in AP-11-NE0.2 with the doubled content of the NEs release decreased from 75 to 65% after 8 h and from 100 to 98% after 24 h (Fig. 8).

### In vitro cytotoxicity

**Fig. 9:**
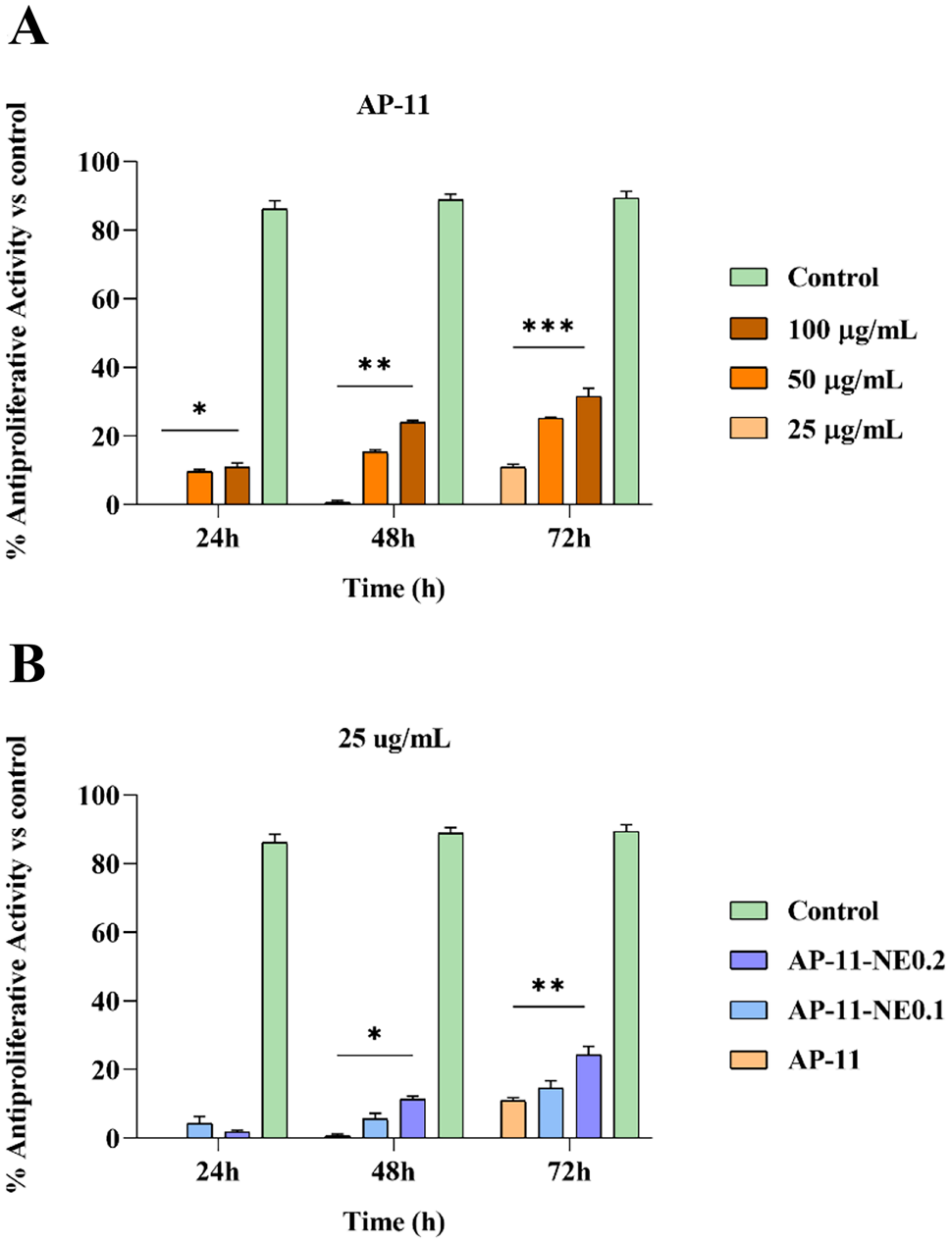
Antiproliferative activity of in situ gel powders assessed by MTT test on HaCaT cells after 24, 48, and 72 h of treatment. Panel **A**: blank formulation AP-11 at different concentrations (25, 50, 100 µg/mL). Panel **B**: nanocomposites AP-11-NE0.1 and AP-11-NE0.2 in comparison with AP-11 at 25 µg/mL. Data are expressed as mean ± SEM. Mercaptopurine (1 µM) was used as a positive control. ***, **, and * denote P < 0.001, P < 0.01, and P < 0.05, respectively, formulations vs control

Since the biocompatibility of any wound dressing is essential to assure the safety of the wounds, the cytotoxicity of the powders on HaCaT cells expressed as an antiproliferative activity through the MTT test was evaluated. The in vitro cell viability assay was conducted by exposing cells to AP-11 and nanocomposites for 24, 48, and 72 h at three different systems concentrations (25, 50, 100 µg/mL). As shown in Fig. 9A, the antiproliferative activity for the blank formulation AP-11 was found to be time and dose-dependent. The increase in powder concentration led to an increase in antiproliferative activity. After 72 h, the powders at the maximum concentration of systems tested (100 µg/mL) exhibited a value of antiproliferative activity slightly higher than 30% that according to ISO 10993–5 is considered cytotoxic [37]. Differently, nanocomposites AP-11-NE0.1 and AP-11-NE0.2, after 72 h, only at 25 µg/mL did not exhibit toxicity (Fig. 9B).

## Discussion

In this work, we developed a tailored hybrid system made of CCM-loaded NEs associated with alginate-pectin matrix to vehicle lipophilic drugs and maximize adherence to the wound. Hydrophobic drugs, once incorporated into NEs, can penetrate efficiently into the skin and through the subcutaneous barrier [38]. However, NEs suspensions are not viscous enough to be directly applied to the wound bed [13]. The composites here produced by spray-drying in the form of microparticles are intended to enhance the contact time at the dressing site prolonging the release of NEs from the hydrophilic matrix.

The first step in the design of adherent nanocomposites was the optimization of NEs loaded with curcumin. NEs obtained via the emulsion phase inversion technique coupled with high stirring energy input presented physicochemical characteristics in accordance with the results reported in a previous study [29]. Then, NEs have been combined with the alginate-pectin matrix to obtain the nanocomposites, and the impact of oil and surfactants on the microparticulate properties, morphology, and size distribution were studied. As reported in the literature, microparticles obtained by spray-drying present different morphologies depending on the features of the initial raw material and processing conditions [39]. In presence of NEs, microparticles did not show the typical morphology of “deflated balloons” but exhibited good sphericity and porous surface due to the global increase of droplet loading with NEs before drying. The encapsulation of NEs in AP did not affect nanocomposites size distribution and surface charge, contrarily to what was previously reported [40]. In fact, they exhibited a mean diameter of around 3.5 µm characterized by a single peak with a span value of less than 2.

To verify possible particles internal structure modifications due to the interaction with the NEs, the thermal behavior of AP-11 microparticles and nanocomposites was investigated by DSC (Fig. 4). In nanocomposites, the characteristic melting and crystallization peaks of NEs were shifted to lower temperatures. This result suggests the presence of NEs in the alginate-pectin matrix as supported by previous work [40], confirming the hypothesis of an interaction between NEs and polymer matrix, also highlighted by the variation of the NEs physicochemical properties after the dissolution of the microparticles in which they were embedded.

Following the physicochemical studies and the analyses of the physical interactions characterizing the nanocomposite, the gelling properties, and fluid permeability were investigated. A rapid hydrogel formation is a crucial characteristic for the in situ gelling powders intended to be applied to the wound with high exudate production. As expected, fluid uptake was strictly correlated to NEs concentration. Compared to the blank formulation, nanocomposites showed lower water uptake (Fig. 5) probably due to the presence of the NEs that physically impaired the movement of the polymer chains during the uncoiling connected to the interaction with water. Modifications such as the addition of hydrophobic nanoparticles in a hydrogel system have been reported to influence the swelling properties of hydrogel. Nanoparticles, replacing the space occupied by water molecules, led to a reduction of water uptake into the polymer structure [41].

Dressing transpiration is another key parameter of wound dressing needed to both prevent an excessive wet condition during wound care and reduce pain removal of the dressing after its use. All hydrogels formed in situ showed WVTR values between 85 and 89 g/m^2^h. These values are in the appropriate range to prevent wound dehydration or occlusion phenomena [42]. Moreover, the amount of water retained by gels even after 72 h with the wound in the dry state is enough to avoid complete gel drying, thus allowing its easy removal at the end of the treatment. WWTR and water loss from hydrogels loaded with NEs were similar to those of the unloaded AP hydrogels (Fig. 6). This similarity suggests that although the presence of a lipophilic portion, nanocomposites could be able to maintain a suitable fluid balance in the wound bed, which might enhance both cellular migration and reepithelization [43]. Concerning the rheological study (Fig. 7), the amplitude sweep test was carried out at 37 °C to simulate the body temperature. With the temperature rising, the particles tend to move disorderly due to a higher Brownian thermal energy, and consequently, while the mobility of macromolecule segments increases, the duration of relaxation processes is reduced [44, 45]. As shown in Fig. 7A the addition of NEs led to an increase of both moduli proportionally to the NEs concentration, whereby the growth of G' is significantly stronger than that of G". This effect is correlated to the influence of the nanoparticles on the mobility of the polymers chains that might determine an increased rigidity of the structure and, as a consequence, stability on the wound [45]. Moreover, as reported in Fig. 7B, the addition of NEs causes a reduction of the loss factor which, when lower than 1, indicates a clear prevalence of gel behavior [46]. This reduction determines an increase in the elasticity of the gels, a very useful property for wound healing devices.

In vitro release studies were carried out in SWF in order to evaluate the ability of the polymer matrix to effectively release NEs loaded with CCM in a sustained manner. This kind of study is generally carried out in sink conditions, where the maximum drug concentration in the bulk fluid does not exceed about 20% of the drug’s solubility [47]. For poorly water-soluble drugs, high dissolution medium volumes are required to maintain a low ratio of particles to medium volume, which however results in a loss of accuracy for the measurement of drug content [48]. In some cases, sink conditions do not guarantee that no saturation occurs, therefore, the release of NEs via curcumin from nanocomposites was performed in non-sink conditions, to avoid underestimation of the drug.

The release profiles of the NEs from nanocomposites (Fig. 8) are due to the coexistence of different conditions, including the porosity of the particles, heterogenous NEs distribution, and the hydrophilic properties of the polymers, which once in contact with SWF diffused into the system, swell and form a gel, causing the system dissolution. After the formation of the gel, which takes place in a few minutes, the dissolved NEs molecules diffuse through the hydrated polymeric network out of the system resulting in their continuous release. In addition (after 8 h), the bioerosion of the polymer might be the main driving force of the sustained release of NEs [47, 49]. CCM, as any other possible drug, loaded into NEs is protected by hydrolysis and various other enzymatic degradations while the environment of the wound would lead to the NEs dissociation and the consequent delivery of CCM [50].

Finally, the biocompatibility of nanocomposites on human keratinocytes (HaCaT) was assessed by monitoring cell metabolic activity (MTT assay) to ensure the safety of the formulations when applied to the wound. HaCaT cells are widely used to test the compatibility of wound dressings as skin consists of different types of cells: keratinocytes, melanocytes, and fibroblasts [51]. In a previous work, we demonstrate that AP-11 powders were not toxic in the range of 0.01–10 µg/mL [24]. In this work, we tested the concentrations ranging from 25 to 100 µg/mL to verify the possibility to use a larger amount of powders on the wound. AP-11 did not show any significant cytotoxicity after 72 h for any of the tested concentrations, except at 100 µg/mL, resulting well tolerated by keratinocytes (Fig. 9A). On the contrary, AP-11-NE0.1 and AP-11-NE0.2 reduced the metabolic activity of cells after 48 h at the 50 and 100 µg/mL in a dose-dependent manner (Fig. S1). At 25 µg/mL and up to 72 h of exposure, both nanocomposites were non cytotoxic (Fig. 9B), indicating the possibility to apply the hydrogels for a prolonged time on the wound bed, thus reducing the patients’ pain caused by frequent removal of the formulations. However, since cellular respiration might be altered by the shielding effect of the hydrogels, further studies will be needed to assess wound healing properties in vivo.

## Conclusions

Nanocomposites combining NEs with alginate-pectin powders intended for the controlled release of lipophilic drugs to be delivered directly to the wound bed were successfully designed. This study proposed a novel approach using a spray-drying process to load nanosystems into in situ gelling microparticles composed of a natural polymeric blend. Nanocomposites could improve wound management thanks to their ability to rapidly form a hydrogel when in contact with simulated wound fluid providing optimal water vapor transmission rate. The combination of polymers and NEs enhanced the elastic properties of the in situ formed gel and lead to the sustained release of the drug-loaded NEs. In vitro cytocompatibility demonstrated that nanocomposites were well tolerated by keratinocytes, allowing a prolonged application of the powder on the wound bed. Globally, the unique combination of NEs loaded into an in situ forming gel microparticulate powder can be considered a promising candidate for novel wound dressings.

## Supplementary Information

Below is the link to the electronic supplementary material.

Supplementary file1 (DOCX 59 KB)

## Data Availability

Not applicable.
