# Supplementary material for: Alginate-pectin microparticles loaded with nanoemulsions as nanocomposites for wound healing
Source: Drug Deliv Transl Res. 2022 Dec 13;13(5):1343–57. doi: 10.1007/s13346-022-01257-9 (PMC10102150; doi:10.1007/s13346-022-01257-9)
Supplement: Supplementary file 1 — Supplementary file1 (DOCX 59 KB) [file 13346_2022_1257_MOESM1_ESM.docx]

Supplementary materials


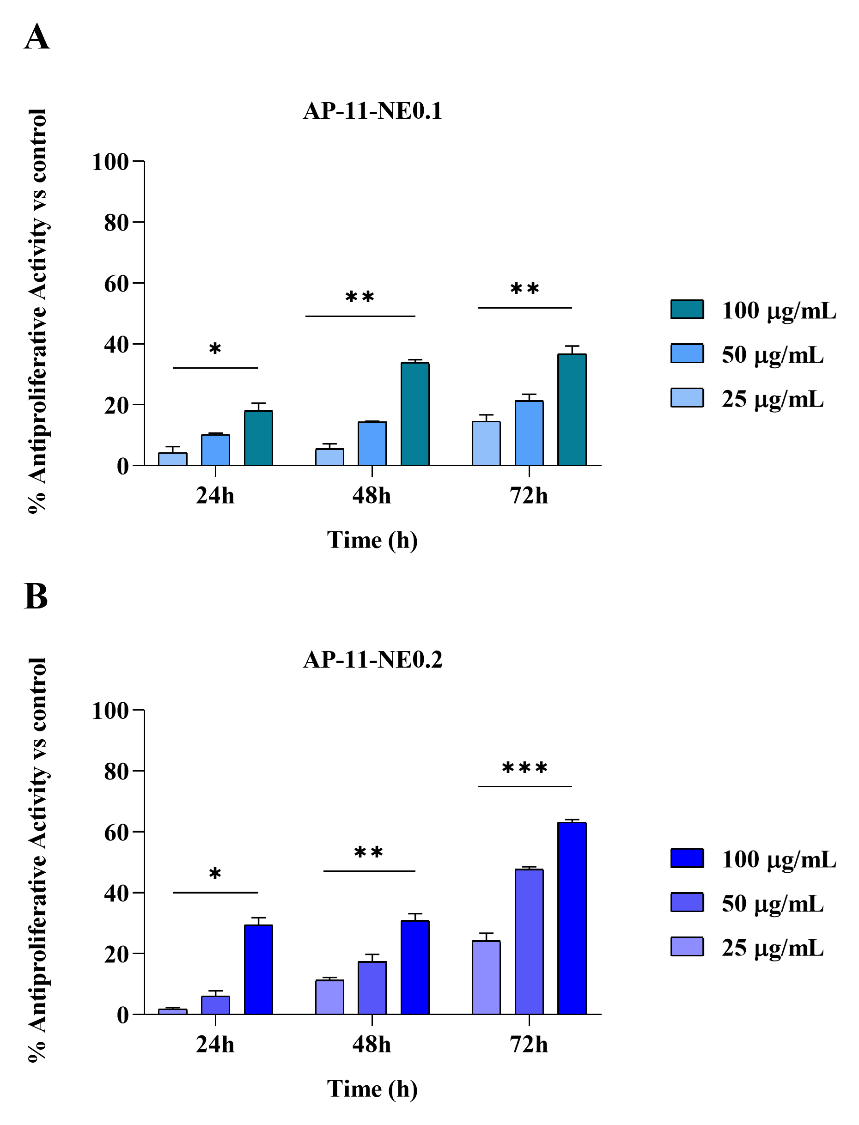


***Fig. S1*** *Anti-proliferative activity of nanocomposites on HaCaT cells, at different concentrations (25-50-100 µg/mL, after 24, 48, and 72 hours of treatment. Panel A:AP-1-NE0.1 Panel B: AP-11-NE0.2. Data are expressed as mean ± S.E.M. Mercaptopurine (1 µM) was used as a positive control. ***, ** and * denote P<0.001, P<0.01 and P<0.05 respectively formulations vs control*
